# Immediate Changes in Perceived Person-Centered Care and Technological Competency Following a One-Day Collective Training Program Among Mongolian Nurse Managers and Nurses Nominated for Managerial Roles: A Pilot Pre–Post Study

**DOI:** 10.3390/nursrep16090320

**Published:** 2026-09-04

**Authors:** Kyoko Osaka, Kaito Onishi, Krishan Soriano, Tetsuya Tanioka, Misao Miyagawa, Masashi Akaike, Yasuhiko Nishioka, Minoru Irahara, Rick Yiu Cho Kwan, Gochoosuren Gankhuyag, Tsogbadrakh Basbish, Taivan Enkhzaya, Otgonbayar Uulensolongo, Enkh-Amgalan Bat-Ulzii, Galmandakh Lkhamaa, Luvsan Munkh-Erdene, Purevdorj Bayasgalan, Badamdorj Oyungoo, Boldbaatar Damdindorj

**Affiliations:** 1 Department of Nursing, Nursing Course of Kochi Medical School, Kochi University, Kochi 783-8505, Japan; osaka@kochi-u.ac.jp; 2 Graduate School of Health Sciences, Tokushima University, Tokushima 770-8509, Japan; kai10.onishi10@gmail.com (K.O.); ksoriano@spup.edu.ph (K.S.); 3 Graduate School of Biomedical Sciences, Tokushima University, Tokushima 770-8509, Japan; akaike.masashi@tokushima-u.ac.jp (M.A.); yasuhiko@tokushima-u.ac.jp (Y.N.); irahara@tokushima-u.ac.jp (M.I.); 4 Faculty of Medicine, School of Health Sciences, Tokushima University, Tokushima 770-8509, Japan; misaomiyagawa2023@gmail.com; 5 School of Nursing, Tung Wah College, Hong Kong 999077, China; rickkwan@twc.edu.hk; 6 Mongolia-Japan Hospital, Mongolian National University of Medical Sciences, Ulaanbaatar 14210, Mongolia; gankhuyag@mnums.edu.mn (G.G.); enkhzaya.t@mnums.edu.mn (T.E.); uulensolongo@mnums.edu.mn (O.U.); lkhamaa@mnums.edu.mn (G.L.); munkherdene@mnums.edu.mn (L.M.-E.); bayasgalan.p@mnums.edu.mn (P.B.); 7 School of Nursing, Mongolian National University of Medical Sciences, Ulaanbaatar 14210, Mongolia; basbish@mnums.edu.mn (T.B.); bat-ulzii@mnums.edu.mn (E.-A.B.-U.); oyungoo@mnums.edu.mn (B.O.); 8 Mongolian National University of Medical Sciences, Ulaanbaatar 14210, Mongolia; damdindorj@mnums.edu.mn

**Keywords:** one-day collective training program, nurse managers, Person-Centered Care, technological competency as caring in nursing theory, pre–post evaluation

## Abstract

**Background/Objectives**: Person-centered care (PCC) and the Technological Competency as Caring in Nursing (TCCN) theory are increasingly emphasized in nursing leadership, but no published study has evaluated a PCC/TCCN training program among Mongolian nurse managers. This uncontrolled, single-group pilot pre–post study examined immediate changes in nurse managers’ self-reported PCC and TCCN perceptions following a one-day collective training program. **Methods**: The program was delivered on 30 October 2025 during the 2nd International Nursing Symposium in Mongolia. Using convenience sampling, hospitals were invited to send nurse managers who were currently in post or senior nurses who had been formally nominated for such a role. Of 50 attendees, 30 met a pre-specified role-eligibility criterion and formed the matched pre–post analytic sample. Participants completed Mongolian versions of the Person-Centered Care Instrument (PCCI) and the Perceived Technological Competency as Caring in Healthcare Providers Instrument (TCCHI) immediately before and after the program. Pre–post differences were assessed with Wilcoxon signed-rank tests, effect sizes for the Wilcoxon signed-rank test (*r* = *z*/*√*N), and Holm–Bonferroni correction across 16 comparisons. **Results**: Total and all concept-level PCCI and TCCHI scores were higher after the program than before it, all 16 comparisons remained significant after correction, and the effect sizes were moderate to large (*r* = 0.41–0.61). The median within-person change was +22.0 points (95% CI 7.0 to 35.5) for the total PCCI score and +24.0 points (95% CI 10.5 to 38.0) for the total TCCHI score (Hodges–Lehmann estimates). **Conclusions**: In this small convenience sample of Mongolian nurse managers and senior nurses formally nominated for managerial roles, self-reported PCCI and TCCHI scores increased immediately after a one-day training program. Because the study used a single-group design without a control group, employed instruments not yet fully validated in Mongolian, and included no follow-up, these findings are preliminary evidence of an association rather than proof of program effectiveness. Larger, controlled studies with follow-up are needed. Trial Registration: TCTR20260513001, registered retrospectively on 13 May 2026, after data collection had been completed.

## 1. Introduction

The global healthcare community widely regards Person-Centered Care (PCC) as the gold standard for quality service delivery, emphasizing individual preferences, autonomy, and values to achieve better health outcomes [1,2]. PCC has emerged as a dominant conceptual framework in nursing and healthcare research since 2017, with its academic and clinical application expanding across diverse healthcare systems worldwide [3]. PCC focuses on the person rather than the illness, respects and actively responds to patients, and ensures that patients’ rights and autonomy are upheld in clinical decision-making [4]. Evidence from systematic reviews further demonstrates that PCC interventions, particularly those integrating self-management support and technology-based approaches, are associated with improvements in patient self-efficacy, reduction in hospitalizations, and enhanced quality of care [5].

In parallel, as healthcare technology advances rapidly, the theory of Technological Competency as Caring in Nursing (TCCN) has become increasingly important [6,7]. This framework asserts that high-quality professional care requires the seamless integration of technical proficiency with genuine empathy and ethical judgment [8]. TCCN redefines technology not as a barrier to human connection but as a vital tool for “knowing the person as caring,” enabling nurses to provide individualized and dignified care even in complex clinical environments [9]. A theoretical analysis of TCCN has demonstrated its applicability across education, research, and clinical practice contexts, underscoring the necessity of grounding the theory in locally adapted frameworks to ensure consistent implementation [10]. Thus, integrating PCC principles with TCCN theory is essential for managing the complexities of modern technological healthcare environments while preserving the core tenets of humanistic care. Although these frameworks are recognized internationally, translating them into clinical practice remains both essential and difficult within the Mongolian healthcare system.

Mongolia, the world’s second-largest landlocked country, has a healthcare system historically shaped by Soviet and Chinese influences. While its capital, Ulaanbaatar, is home to nearly half of the population and has well-developed medical facilities, rural and remote regions—including the nomadic Gobi area—face significant disparities in healthcare access [11]. Although Mongolia’s national nurse-to-population ratio (42.14 per 10,000) [12] is marginally above the global average [13], it remains well below the OECD average [14] and neighboring Asian systems such as Japan [15], and this national figure masks substantial urban–rural maldistribution and a persistent lack of professional autonomy among frontline nursing staff [11,16].

These systemic constraints, combined with heavy workloads, often force nurses to prioritize task-oriented care over PCC, while limited leadership-development opportunities continue to hinder nurses’ capacity to advance into management roles [17]. Similar structural barriers to nursing leadership development—including restricted career progression for female nurses and limited educational infrastructure—are documented across other low- and middle-income countries (LMICs) [18,19,20].

In such resource-constrained contexts, current in-service education in Mongolia remains fragmented, often lacking the systematic structure, interprofessional coordination, and measurable evaluation methods needed to bridge theoretical concepts of PCC and TCCN with daily practice [21,22]. Effective implementation of these frameworks requires integration of education with evidence-based practice and quality improvement [23,24,25]. To date, however, no published study has evaluated an educational program grounded specifically in PCC and TCCN theory among Mongolian nurse managers, and the Mongolian-language instruments used to assess these constructs have not previously been applied in this population. This represents the specific knowledge gap that the present pilot study was designed to address.

Therefore, the objective of this pilot study was to examine immediate changes in participants’ self-reported PCCI and TCCHI scores following the program, using the newly adapted Mongolian versions of the Person-Centered Care Instrument for Healthcare Providers (PCCI) [26] and the Perceived Technological Competency as Caring in Healthcare Providers Instrument (TCCHI) [27]. On the basis of the program content and of previous reports on short educational interventions, we anticipated that participants’ self-reported PCCI and TCCHI scores would be higher immediately after the program than before it. Because the trial was registered retrospectively, this expectation was not lodged in a public registry before data collection and is therefore stated here as an analytic expectation rather than as a formally pre-specified hypothesis.

## 2. Materials and Methods

### 2.1. Study Design and Participants

This study employed a single-group, uncontrolled pre–post design to examine immediate changes in self-reported perceptions following an in-service education program. Because there was no comparison group, and because the post-intervention assessment was conducted immediately after the program, the design cannot establish the effectiveness of the intervention or demonstrate changes in professional competence, clinical behavior, or patient outcomes. Reporting of this study was guided by the Transparent Reporting of Evaluations with Nonrandomized Designs (TREND) statement [28]. Items that could not be fully addressed given the pilot nature of the study (e.g., long-term follow-up) are identified as limitations in Section 4.4.

The educational program was embedded as a core interactive session within the 2nd International Nursing Symposium, held on 29 and 30 October 2025; the program evaluated in this study was delivered on the second day, 30 October 2025. This symposium was co-organized in collaboration with the Mongolian National University of Medical Sciences (MNUMS) and the Japan International Cooperation Agency (JICA) project aimed at strengthening hospital management capacities in Mongolia and attracted more than 100 healthcare professionals, including policymakers from the Ministry of Health, academic faculty, and clinical leaders nationwide.

To recruit participants for this specific intervention, official invitations were distributed via the nursing departments of the Mongolia-Japan Hospital and MNUMS to nursing directors at various clinical institutions across Mongolia (primarily located in the Ulaanbaatar metropolitan area), using a convenience sampling method. Invitations explicitly requested that each participating hospital send currently practicing nurse managers (or, where applicable, senior nurses formally nominated for an imminent managerial role). Eligibility was defined by role: an attendee had to hold one of the following nursing management positions—charge nurse, ward nurse manager, deputy director of nursing, or director of nursing—or to have been formally nominated by their hospital for such a position. Before the symposium, the Mongolia-Japan Hospital, MNUMS, sent an official letter (ref. 05/763, dated 10 October 2025) to the directors and deputy directors of nursing of the invited healthcare organizations, requesting that they nominate members of their nursing management teams to attend; attendees were therefore nominated by their own institutions, and nominee details were returned to the organizers by 22 October 2025. The letter was sent to the nursing directorates of 53 healthcare organizations in both the Ulaanbaatar metropolitan area and provincial or rural areas. Thirty-six of these organizations nominated participants, and 17 did not, either declining, not responding, or attending only the first day of the symposium. Across the 36 participating organizations, 20 nominated one nurse, 14 nominated two, one provincial regional diagnostic and treatment center nominated four, and the Mongolia-Japan Hospital nominated six, giving 58 nominees in total, of whom 38 were based in Ulaanbaatar and 20 in provincial areas. The number of individual nurses approached within each organization, and the number who declined their institution’s nomination, was recorded by the institutions themselves and is not available to us. This role-based eligibility criterion—holding, or being formally nominated for, a nurse-manager position—was defined before the training program and data collection began and was the sole basis for inclusion in the analytic sample.

Fifty of the 58 nominees attended the one-day training program evaluated in this study. However, 20 attendees were identified on-site as ineligible, because they neither held a managerial position nor had been formally nominated for one. Because the organizers relied on the institutional nomination process and the job titles of individual nominees were not communicated to them in advance, eligibility could not be established before the day of the program itself. Verification was therefore carried out at registration, before the program began and before the pre-intervention survey: a member of the research team inspected each attendee’s employee identification card, which is issued by the employing hospital and states the holder’s position. The questionnaires were nevertheless administered to every attendee present, because the educational session itself was open to all symposium participants and no one was turned away. The role-eligibility criterion was then applied at the analysis stage. Consequently, these 20 individuals were excluded on the basis of the predefined role-eligibility criterion, not on the basis of their questionnaire scores.

The final matched pre–post analytic sample comprised 30 eligible participants: 28 current nurse managers and two senior nurses formally nominated for managerial roles. Throughout this article, the term “nurse managers” denotes this combined study population. The two groups were not analyzed separately because the study was not powered for subgroup comparison; a sensitivity analysis excluding the two nominated senior nurses is reported in Section 3.2 (see Supplementary Table S1). The 20 excluded attendees did complete the questionnaires, which were administered to everyone present on the day of the program, but their responses were not entered into the analytic dataset and were not used in any analysis reported here.

Because eligibility could only be verified on the day of the program rather than at the point of nomination, selection bias cannot be fully ruled out. The number of individual nurses invited within each hospital, and the number who declined, could not be reconstructed, because nomination was handled by the participating institutions rather than by the organizers. Both issues are acknowledged as limitations (Section 4.4).

The analytic sample size (*n* = 30) was determined by the number of eligible attendees available at this single pilot session rather than by an a priori power calculation. Given the pilot, hypothesis-generating nature of this study, a retrospective post hoc power calculation was not performed.

### 2.2. One-Day Collective Training Program

The program was designed to enhance practical competence in PCC and TCCN. The program was delivered as a one-day session lasting 7.5 h in total, comprising 330 min (5.5 h) of scheduled lectures and participatory workshop activities, 30 min for each of the pre- and post-intervention surveys, and a one-hour lunch break (Table 1). The theoretical core integrated the PCC framework, its eight core concepts, and TCCN theory [2,6,7]. It also incorporated Carper’s [29] four fundamental patterns of knowing in nursing (empirical, ethical, personal, and aesthetic) and concepts of technological competency as caring in healthcare providers [30]. The educational strategy drew on hierarchical deployment methods [31] and an evaluation framework based on the Kirkpatrick model [32]. The program was delivered by the Japanese research team, which included professors specializing in nursing science and TCCN theory, thereby ensuring expert-led delivery of the curriculum. All sessions were conducted in Mongolian. Lectures were delivered in Japanese and rendered into Mongolian by a professional interpreter fluent in Japanese and in medical terminology. Because consecutive rather than simultaneous interpretation was used, roughly half of each scheduled lecture slot shown in Table 1 was occupied by interpretation, so the effective time devoted to lecture content was approximately half of the time listed. Workshop activities were conducted in small groups of approximately eight participants each, facilitated in Mongolian.

Although participants were nominated by their respective healthcare institutions to attend the educational symposium, participation in the research evaluation was strictly voluntary and distinct from event attendance. Prior to baseline data collection, written informed consent was obtained on-site by the research team. Participants were explicitly informed that choosing not to complete the questionnaires was entirely voluntary, that responses would be processed anonymously, and that non-participation would have no negative impact on their symposium attendance, professional standing, or institutional evaluation. As this pilot study involved only a classroom-based educational program and the completion of self-report questionnaires, it was considered minimal-risk; no adverse or unintended events were reported by participants or observed by the research team during or after the program, and no formal adverse-event monitoring protocol beyond informal observation by the on-site research team was implemented. The specific structure, themes, and content of the program are summarized in Table 1. The lecture materials, slides, and workshop facilitation guide used in this program are not included in this publication and remain the copyright of the authors. They may be made available by the corresponding author on reasonable request for non-commercial academic or educational use, subject to a written agreement covering attribution and restrictions on redistribution, adaptation, and translation. Requests may be declined or deferred where the materials are being prepared for separate publication or development as a teaching resource.

### 2.3. Measures

Data were collected using a web-based questionnaire. Both surveys were completed individually on site under the supervision of the research team: the pre-intervention survey in a 30 min slot immediately before the first lecture, and the post-intervention survey in a 30 min slot immediately after the final session. At enrolment, each participant was assigned a code number carrying no identifying information, and this code was the only identifier entered into the questionnaire. Pre- and post-intervention responses were matched by this code (linkable anonymization); no names or other directly identifying information were collected [34]. The questionnaire included demographic variables—specifically capturing participants’ age, gender, total years of clinical nursing experience, duration of experience in managerial roles, highest level of nursing education achieved, and current institutional position. In addition, the core variables described below were rated on a 6-point Likert scale (1 = “Strongly Disagree” to 6 = “Strongly Agree”):

*Translation and cultural adaptation of the instruments.*

Both instruments were originally developed in English and were translated into Mongolian for use in this study. Forward translation into Mongolian and independent back-translation into English were performed by three professional Mongolian translators fluent in both Japanese and English. Each back-translation was compared with the source version; items whose meaning had shifted were identified, the Mongolian wording was revised, and the material was re-translated. This cycle was repeated until the back-translated and source versions were judged to convey equivalent meaning. Discrepancies arising during this process were reconciled by a JICA consultant who is a co-investigator of the present study. The resulting Mongolian drafts were then reviewed for conceptual, semantic, and cultural equivalence by a panel of six experts, including the developer of the original instruments, and were pre-tested for comprehensibility with five Mongolian nurses, whose comments were incorporated into the final versions. No formal psychometric evaluation of the Mongolian versions (e.g., factor analysis, criterion-related validity, or test–retest reliability) has yet been undertaken. Accordingly, the reliability coefficients reported in Section 3.2 describe the behavior of the translated instruments in the present sample only, and are not presented as evidence that the Mongolian versions have been validated.

PCCI [26]: The PCCI was employed to evaluate the participants’ perceived competence in delivering PCC across interdisciplinary contexts. This instrument consists of 37 items structured into eight concepts: (1) Respect and Empathy, (2) Partnership and Trust, (3) Individualization and Consideration for Diversity, (4) Shared Decision-Making, (5) Emotional and Psychological Support, (6) Comprehensive Care and Holistic Perspective, (7) Effective Information Sharing with Care Recipients, and (8) Flexible Care. Each item is rated on a 6-point Likert scale (ranging from 1 = “Strongly Disagree” to 6 = “Strongly Agree”), yielding a total potential score ranging from 37 to 222 points. Higher total scores reflect a higher level of professional competence and readiness in delivering personalized, dignified, and flexible care.

The original English PCCI was developed through a two-round modified Delphi procedure with a panel of ten interdisciplinary healthcare experts. For the final 37-item version, the scale-level content validity index calculated by the averaging method (S-CVI/Ave) was 0.65, and the index calculated by the universal agreement method (S-CVI/UA) was 0.22. Both values fall below the thresholds conventionally recommended for established instruments (S-CVI/Ave ≥ 0.80–0.90). The developers attributed the low S-CVI/UA in particular to the interdisciplinary composition of the panel: experts from nursing, medicine, and physiotherapy weighted the importance of individual items differently according to their own clinical context, so that only 3 of the 37 items were rated as extremely important by every panel member, and they identified exploratory and confirmatory factor analysis as the necessary next steps.

TCCHI [27]: The TCCHI was used to measure nurses’ perceptions of integrating technical proficiency with compassion and empathy, extending Locsin’s theoretical paradigm for multidisciplinary use. The instrument comprises 38 items categorized into six refined concepts: (1) supporting healthcare professionals’ growth, (2) building trusting relationships with patients, (3) providing PCC, (4) enhancing the physical and emotional comfort of patients, (5) promoting patient learning and growth, and (6) engaging in ethico-moral practices. Respondents rate each item on the same 6-point Likert scale (1 to 6), resulting in a total score range of 38 to 228 points. A higher cumulative score indicates a higher perceived capacity to utilize healthcare technologies not as barriers, but as vital tools for deeply knowing and caring for patients as whole persons. Each concept score is the simple sum of the scores of the items belonging to that concept, each item being rated from 1 to 6. The number of items and the possible score range for all 14 concepts and for the two total scores are as follows: for the PCCI, concept 1, 4 items (4–24); concept 2, 5 items (5–30); concept 3, 4 items (4–24); concept 4, 6 items (6–36); concept 5, 4 items (4–24); concept 6, 4 items (4–24); concept 7, 6 items (6–36); and concept 8, 4 items (4–24), giving a total score range of 37 to 222; and for the TCCHI, concept 1, 6 items (6–36); concept 2, 7 items (7–42); concept 3, 6 items (6–36); concept 4, 6 items (6–36); concept 5, 6 items (6–36); and concept 6, 7 items (7–42), giving a total score range of 38 to 228. All items were mandatory in the web-based form; no missing item responses occurred, and no imputation was required.

Content validity for the original English TCCHI was evaluated through a two-round modified Delphi method with ten multidisciplinary healthcare experts. Of the initial 67 items, 38 were retained after meeting the item-level retention criterion (I-CVI ≥ 0.80, range 0.80–0.90), and rating stability across rounds was acceptable (*p* > 0.05). However, inter-rater reliability was moderate (ICC = 0.50), and the scale-level content validity indices were low (S-CVI/Ave = 0.55, S-CVI/UA = 0.02). As with the PCCI, the original developers noted that these values should be interpreted within the context of preliminary validation and the instrument’s novel, multidisciplinary focus. We therefore describe the content validity of the TCCHI, like that of the PCCI, as preliminary rather than as established.

This evidence only pertains to the original English versions of the instruments. A separate content-validity evaluation was not conducted for the Mongolian version used in this study.

### 2.4. Statistical Analysis

Statistical analysis was conducted using jamovi statistical software, version 2.4.11.0 (The jamovi project, Sydney, NSW, Australia) [35]. The level of statistical significance was set at *p* < 0.05.

Descriptive Statistics: Descriptive statistics and frequency distributions were used to summarize the demographic profile of the participants. Continuous demographic variables (e.g., age and years of nursing experience) were summarized using medians and interquartile ranges (IQRs) to accommodate potential distributional skewness in a small sample.

Reliability Analysis: To evaluate the internal consistency of the translated instruments within the present pilot sample (*n* = 30), McDonald’s omega (*ω*) and Cronbach’s alpha (α) coefficients were computed for the total scale scores of both the PCCI and TCCHI. Values of ω ≥ 0.70 and α ≥ 0.70 were considered to indicate acceptable sample-specific internal consistency.

Pre–Post Comparison: Normality of the data was assessed using the Shapiro–Wilk test. Although several variables did not violate the assumption of normality (*p* > 0.05), the non-parametric Wilcoxon signed-rank test was used for all pre- and post-intervention comparisons. This conservative approach was chosen to ensure statistical robustness, given the small paired sample size (*n* = 30) and the ordinal nature of Likert-scale items. For each comparison, the effect size *r* for the Wilcoxon signed-rank test was calculated as *r* = *z/√N*, where z is the standardized test statistic and N is the number of paired observations (*n* = 30), and was interpreted using conventional benchmarks (*r* ≈ 0.10, small; 0.30, medium; ≥0.50, large) [36,37]. Because 16 comparisons were conducted across the total and concept-level scores of the two instruments (9 for PCCI, 7 for TCCHI), a Holm–Bonferroni step-down correction was applied across all 16 comparisons to control the family-wise error rate; both the original and Holm-adjusted *p*-values are reported [38]. The total PCCI score and the total TCCHI score were treated as the primary outcomes of this pilot study; the 14 concept-level comparisons were secondary and exploratory, and are reported for completeness. All 16 comparisons were nevertheless included in a single Holm–Bonferroni family so that the family-wise error rate is controlled across the full set. Because the trial was registered only after data collection had been completed, this hierarchy of outcomes was not formally pre-specified in a public registry entry and should therefore be regarded as an analytic, rather than a confirmatory, designation. Nonetheless, this designation of the total PCCI and total TCCHI scores as the primary outcomes was determined prior to the training program, at the time the study protocol was submitted for institutional ethical review at Tokushima University (approved on 30 June 2025, approval no. 4708; see Section 2.5), and not after data collection or analysis. The matched-pairs rank-biserial correlation was not calculated in this study. In addition, a sensitivity analysis was conducted in which the two participants who had been formally nominated for, rather than currently holding, a managerial position were excluded (*n* = 28). This analysis was not pre-specified and was carried out in response to peer review. For the two primary outcomes, the within-person change was additionally summarized using the Hodges–Lehmann median difference with a distribution-free 95% confidence interval (CI).

### 2.5. Institutional Ethical Approval and Trial Registration

This study was approved by the Ethics Committee of Tokushima University Hospital on 30 June 2025 (approval no. 4708; title: “Measuring the effectiveness of in-service education for providing high-quality healthcare services”). The Mongolian-language versions of the two instruments had been developed under a separate approval from the same committee (approval no. 4591, 30 September 2024). The study protocol was also reviewed and approved by the Clinical Research Subcommittee of the Mongolia-Japan Hospital, Mongolian National University of Medical Sciences (MNUMS) at its meeting on 25 October 2025 (approval no. A/08-2). The 2nd International Nursing Symposium was held on 29 and 30 October 2025, and the training program evaluated here, together with both data collections, took place on 30 October 2025. Both approvals covering the present study (nos. 4708 and A/08-2) therefore preceded the start of the intervention and of data collection. Because this study was designed as a small-scale pilot educational intervention embedded within an international symposium, the research team initially prioritized securing these institutional ethical approvals and managing complex international logistics before completing trial registration; as a result, the trial was retrospectively registered with the Thai Clinical Trials Registry (TCTR20260513001) on 13 May 2026, after participant recruitment, the intervention, and data collection had already been completed in October 2025. This retrospective registration is acknowledged as a study limitation (Section 4.4). Full trial registration details and institutional review board statements are provided at the end of this manuscript.

## 3. Results

### 3.1. Participant Flow and Demographic Profile of Nurse Managers

**Figure 1:**
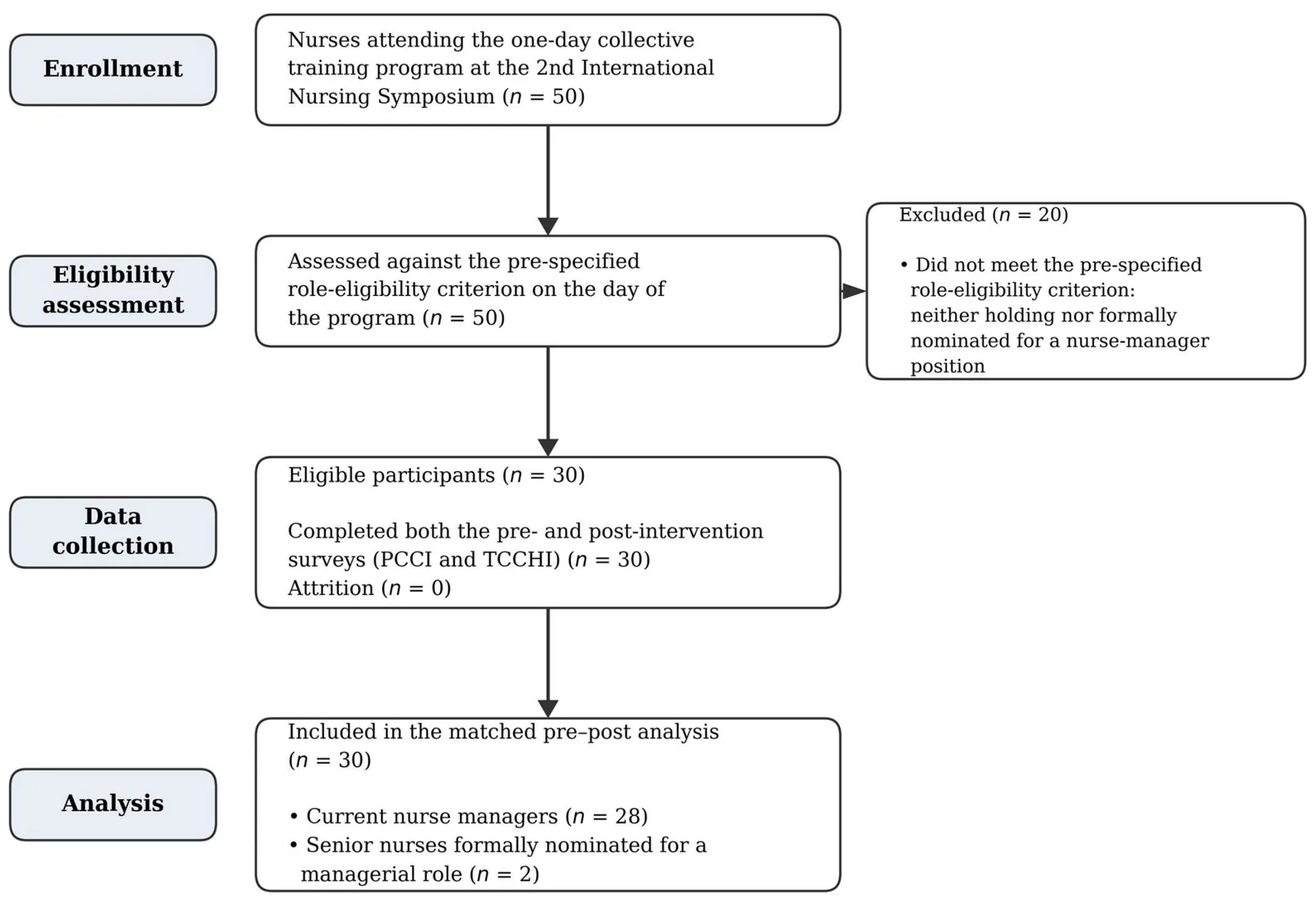
Participant flow diagram. PCCI = Person-Centered Care Instrument for Healthcare Providers; TCCHI = Perceived Technological Competency as Caring in Healthcare Providers Instrument.

As illustrated in the participant flow diagram (Figure 1), 50 nursing professionals attended the one-day program; of these, 20 were excluded because, on assessment on the day of the program, they did not meet the pre-specified role-eligibility criterion (current nurse-manager position or formal nomination for one). The remaining 30 participants were included in the matched pre–post analysis, with complete pre- and post-intervention data and no attrition in this analytic sample. As stated in Section 2.1, the questionnaires completed by these 20 attendees were not used in any analysis. All 30 participants in the analytic sample attended the training program in its entirety, from the pre-intervention survey through the post-intervention survey, with no early departures.

The demographic profile of these 30 participants is summarized in Table 2. All participants were female, comprising 28 current nurse managers and two senior nurses formally nominated for managerial roles. These two senior nurses were officially nominated by their respective hospitals based on their demonstrated administrative competencies and leadership potential, as they are expected to assume pivotal nurse managerial roles in the near future.

The most frequent age group was 40–49 years (36.7%), and most of the remaining participants were distributed equally between the 30–39 and 50–59 year age groups (30.0% each). Regarding professional experience, the majority had substantial clinical backgrounds: 40.0% reported 10–19 years of experience, while 53.4% had 20 years or more. Notably, the participants were highly educated, with over half (53.3%) holding a Master’s degree, followed by those with a Bachelor’s degree (23.3%) and a Diploma (23.3%).

All 28 serving nurse managers held one of the four qualifying nursing management positions defined in Section 2.1. In Table 2 they are listed under their principal institutional designation: Chief Nursing Officers (CNOs)/Directors (5/30, 16.7%), Deputy Directors/Assistant CNOs (4/30, 13.3%), Head Nurses/Nurse Managers (9/30, 30.0%), Charge Nurses/Supervisors (1/30, 3.3%), In-Service Nurse Educators (3/30, 10.0%) and Nurse Specialists (6/30, 20.0%). The last two designations identify nurse managers who, in addition to holding one of the four qualifying positions, carry designated in-service education or clinical-specialist responsibilities; they are listed under those designations so that each participant is counted once. The remaining two participants (2/30, 6.7%) were Senior Nurses who had been formally nominated by their institutions for imminent appointment to a managerial position. Geographically, participants were recruited from healthcare facilities in both the Ulaanbaatar metropolitan area (*n* = 17, 56.7%) and non-metropolitan/rural regions (*n* = 13, 43.3%).

### 3.2. Comparison of PCCI and TCCHI Scores Before and After the Program

In the present sample (*n* = 30), internal consistency was acceptable for the Mongolian versions of both the PCCI (*ω* = 0.979, *α* = 0.969) and the TCCHI (*ω* = 0.968, *α* = 0.790). These coefficients describe internal consistency within this sample only and are not evidence that the Mongolian versions have been validated. The gap between ω and α for the TCCHI (0.968 vs. 0.790) indicates that its items differ appreciably in how strongly they relate to the underlying construct, so α understates the internal consistency of the scale; this pattern further underlines the need for a full psychometric evaluation of the Mongolian version.

Table 3 shows the comparison of participants’ self-reported PCCI and TCCHI scores before and after the one-day collective training program, together with effect sizes (*r*) and Holm–Bonferroni-adjusted *p*-values. Post-intervention scores were statistically significantly higher than pre-intervention scores for the total score and all concepts of both instruments, and all 16 comparisons remained significant after correction for multiple comparisons (Holm-adjusted *p* < 0.05).

#### 3.2.1. Person-Centered Care Instrument for Healthcare Providers (PCCI)

The total PCCI score was higher after the intervention than before it, rising from a pre-program median of 177.5 (IQR: 24.5) to a post-program median of 188.0 (IQR: 32.25) (*z* = −2.98, *p* = 0.003, Holm-adjusted *p* = 0.023, *r* = 0.54). The median within-person change in the total PCCI score was +22.0 points (Hodges–Lehmann estimate; 95% CI 7.0 to 35.5). All eight concepts of the PCCI showed statistically significant improvements (Holm-adjusted *p* < 0.05 for all).

Effect sizes were moderate to large across all PCCI concepts. The largest effect size was observed for concept 3 (Individualization/Diversity, *r* = 0.61, *z* = −3.32), followed by concept 8 (Flexible Care, *r* = 0.58), concept 2 (Partnership and Trust, *r* = 0.56), concept 5 (Emotional and Psychological Support, *r* = 0.55), concept 6 (Comprehensive Care/Holistic Perspective, *r* = 0.51), concept 1 (Respect and Empathy, *r* = 0.47), concept 4 (Shared Decision-Making, *r* = 0.45), and concept 7 (Effective Information Sharing, *r* = 0.45) (see Table 3).

For concept 3, the pre- and post-intervention medians were identical (20.0 vs. 20.0). The Wilcoxon signed-rank test evaluates the magnitude and direction of individual paired differences rather than group medians alone; a significant result with an unchanged median therefore indicates a systematic pattern of within-person score increases that the median does not capture.

#### 3.2.2. Results of the Perceived Technological Competency as Caring in Healthcare Providers Instrument (TCCHI)

The total TCCHI score was higher after the intervention than before it, rising from a pre-program median of 172.5 (IQR: 28.5) to a post-program median of 192.0 (IQR: 33.0) (*z* = −3.17, *p* = 0.002, Holm-adjusted *p* = 0.019, *r* = 0.58). The median within-person change in the total TCCHI score was +24.0 points (Hodges–Lehmann estimate; 95% CI 10.5 to 38.0). All six concepts of the TCCHI showed statistically significant differences (Holm-adjusted *p* < 0.05 for all).

The largest effect sizes among the TCCHI concepts were observed for concept 5 (Promoting patient learning/growth, *r* = 0.60) and concept 3 (Providing PCC through appropriate use of technology, *r* = 0.59), followed by concept 1 (Promoting self-growth, *r* = 0.58), concept 4 (Enhancing physical/emotional comfort, *r* = 0.56), concept 2 (Building trust relationship, *r* = 0.54), and concept 6 (Engaging in ethico-moral practice, *r* = 0.41) (see Table 3).

In all 16 comparisons, most participants showed score increases after the intervention. Specifically, for the PCCI total score, 21 participants (70.0%) increased, 8 (26.7%) decreased, and 1 (3.3%) remained unchanged. Similarly, for the TCCHI total score, 22 participants (73.3%) increased, 7 (23.3%) decreased, and 1 (3.3%) remained unchanged. The detailed distribution of increased, decreased, and unchanged scores for all total and concept-level metrics is presented in Table 3. In a sensitivity analysis excluding the two participants who had been formally nominated for, rather than currently holding, a managerial position (*n* = 28), all 16 comparisons remained statistically significant after Holm–Bonferroni correction (all adjusted *p* ≤ 0.019), and the effect sizes ranged from 0.48 to 0.66. The direction and the overall pattern of the findings were therefore unchanged; the full results of this analysis are presented in Supplementary Table S1.

## 4. Discussion

### 4.1. Summary of Key Findings

This uncontrolled pilot pre–post study examined immediate changes in self-reported PCC and TCCN perceptions among 30 Mongolian nurse managers following a one-day collective training program. Total and all concept-level PCCI and TCCHI scores were higher after the program than before it, and all 16 comparisons remained statistically significant after Holm–Bonferroni correction for multiple comparisons, with moderate to large effect sizes. These findings are consistent with the expectation stated in the Introduction that self-reported scores would be higher immediately after the intervention; as noted there, that expectation was not formally pre-specified before data collection. Because the study used a single-group design without a control group and assessed outcomes only immediately after the program, these results should be interpreted as evidence of an immediate, statistically significant association between participation in the program and higher self-reported PCCI and TCCHI scores. They should not be interpreted as evidence that the program caused changes in professional competence, managerial behavior, or patient outcomes, or that any such changes would be sustained.

### 4.2. Comparison with Previous Studies

The pattern of change observed here is broadly consistent with prior reports that short, structured educational interventions grounded in PCC and TCCN theory are associated with improved self-reported PCC-related perceptions and attitudes among nurses, including in resource-constrained settings [39,40]. The concept-level results are also consistent with earlier work among Japanese nurse managers, in which TCCN-focused education was associated with greater self-reported awareness of technology as a means of “knowing the person,” and with evidence that management-level education may be an important entry point for organizational change [41]. The present pilot study extends this literature by applying newly adapted Mongolian versions of the PCCI and TCCHI in a Mongolian nurse-manager sample for the first time in a setting where in-service education on these frameworks has not previously been evaluated.

### 4.3. Cautious Interpretation of the Findings

Several methodological features of this pilot study limit how the observed score increases should be interpreted. First, because there was no untrained control group, we cannot rule out that some or all of the observed change reflects factors other than the training content itself, including familiarity with the questionnaire on second administration (testing effects), a tendency to provide more socially desirable responses after an educational event emphasizing PCC and caring values, or participants’ awareness of the study’s aims (response-shift or demand-characteristic effects). Second, because participants completed both assessments on the same day as the intervention, and because the staff who facilitated the training were also members of the research team, blinding of participants and trainers was not feasible; this is common in single-day educational evaluations but should be considered when weighing the findings. Third, an increase in self-reported PCCI and TCCHI scores reflects a change in participants’ perceptions; it does not, by itself, demonstrate a change in participants’ underlying knowledge, observable competencies, professional behavior, or the quality of care ultimately experienced by patients. These are distinct constructs, and this pilot study measured only the first of them. For these reasons, we interpret the present findings as preliminary evidence that self-reported PCC and TCCN perceptions increased immediately following the training, rather than as evidence of the program’s effectiveness in a broader sense.

Regarding the participants whose scores were unchanged or decreased following the program (30.0% showing decreased or unchanged scores for the PCCI total, and 26.7% for the TCCHI total), two plausible educational mechanisms warrant consideration. First, unchanged scores in some participants may reflect ceiling effects: those whose pre-intervention perceptions were already very high had limited scope for further increases. Second, a post-intervention score decrease does not necessarily indicate a negative educational outcome; rather, it may represent a response-shift phenomenon. Through intensive exposure to the PCC and TCCN frameworks during the training, participants may have recalibrated their internal standards and reflected more critically on their own leadership practice. Both explanations are offered as possible interpretations of the observed pattern; the present design provides no means of establishing which, if either, actually operated. Future longitudinal studies incorporating qualitative evaluations are warranted to explore individual-level trajectories and determine specific learner characteristics associated with varying response patterns.

It should also be noted that statistical significance, as reported here, indicates that the observed pre–post differences were unlikely to have arisen by chance; it does not, by itself, establish that these changes are clinically or educationally meaningful. Judgments about practical significance would require pre-specified minimal important differences for the PCCI and TCCHI, which have not yet been established for these instruments.

With these caveats in mind, the pattern of moderate to large effect sizes across all concepts of both instruments, together with the program’s explicit grounding in PCC and TCCN theory and its participatory, workshop-based design, is consistent with—but does not prove—a genuine, program-related shift in participants’ self-reported orientation toward person-centered, technologically integrated care. Whether the present findings represent a meaningful step toward the kind of leadership-level cultural change [20,23] that has been proposed as important for embedding PCC and TCCN in institutional practice is a question for future, controlled research rather than a conclusion supported by the present design. It should be emphasized that these effect sizes describe the magnitude and consistency of within-person rank changes in a small, uncontrolled sample assessed immediately after a single session; a statistically moderate or large effect size in this context should not be read as demonstrating an educational or clinically meaningful effect of comparable magnitude.

### 4.4. Limitations and Future Directions

This pilot study has several limitations that should be considered when interpreting its findings. First, the single-group, uncontrolled pre–post design, with outcome assessment immediately after the intervention, does not allow us to attribute the observed score changes to the training program itself, to rule out testing effects, social desirability, or response-shift bias, or to determine whether any changes were sustained beyond the day of the program; participant and trainer blinding was also not feasible. Second, although the role-eligibility criterion for the analytic sample (current or prospective nurse-manager status) was defined before the training and data collection, individual eligibility could only be confirmed on the day of the program, and a full accounting of the number of individuals invited within each hospital and of those who declined was not available; this leaves some residual uncertainty about selection processes that we cannot fully resolve with the available records. Third, the sample was small, recruited by convenience sampling from hospitals that were willing and able to release staff for the symposium, entirely female, and included a relatively high proportion of participants with graduate-level education; the findings therefore apply to this specific group of participants and institutions and should not be generalized to all Mongolian nurse managers or to other resource-limited settings without further evaluation. Fourth, the Mongolian versions of the PCCI and TCCHI have not yet undergone complete psychometric validation (e.g., construct or criterion validity); because the broader measurement properties of these translated instruments have not been fully established, this adds uncertainty to the interpretation of the observed score changes. Fifth, the trial was registered retrospectively, after recruitment and data collection had already been completed, which we acknowledge as a transparency limitation. Finally, this study measured only self-reported perceptions and did not assess knowledge, observable competencies, managerial behavior, or patient-level outcomes.

Future research should build on this pilot work with adequately powered, controlled studies that include an untrained comparison group, follow-up assessment at multiple time points (e.g., one, three, and six months) to evaluate whether perceptual changes are sustained, formal psychometric validation of the Mongolian PCCI and TCCHI, and objective measures of managerial practice or patient-relevant outcomes. Qualitative exploration of participants’ experiences of the program may also help identify Mongolia-specific facilitators and barriers to embedding PCC and TCCN principles in nursing leadership practice.

## 5. Conclusions

In this small convenience sample of Mongolian nurse managers and senior nurses formally nominated for managerial roles, self-reported PCCI and TCCHI scores increased immediately after a one-day training program, with moderate to large effect sizes; all 16 comparisons remained statistically significant after correction for multiple comparisons. Because the study lacked a control group, used Mongolian instrument versions that have not yet undergone complete psychometric validation, and included no follow-up beyond the day of the intervention, these findings should be considered preliminary. Larger, controlled studies with follow-up assessments are needed to determine whether these perceptual changes are sustained and whether they translate into managerial practice or patient-care outcomes.

## Figures and Tables

**Table 1:** Structure and content of the one-day collective training program.

Time	Session	Key Themes and Content Overview
Morning (30 min)	Pre-Evaluation Survey	Pre-intervention assessment using PCCI and TCCHI.
	Lecture Session	Theme: Understanding High-Quality Healthcare Services Based on PCC and Caring Concepts.
60 min	Lecture 1	What is High-Quality Healthcare Service Based on PCC and Caring? Overview of PCC’s 8 core concepts and TCCN theory on the harmony of technology and caring.
40 min	Lecture 2	Required Initiatives for High-Quality Healthcare Service at the Mongolia-Japan Hospital: Introduction of hospital practice examples, quality definitions based on ISO 9001 [33] (Patient Safety, Timely manner, PCC, etc.), and their evaluation system.
Afternoon	Workshop Session	Theme: Concepts of High-Quality Healthcare Service Based on PCC and Caring and In-Service Education.
50 min	Lecture 3	In-Service Education for Practice: Content and evaluation methods for in-service education based on TCCN theory, and hierarchical deployment methods for manager and staff programs.
60 min	Group Discussion	Theme: “In-Service Education for Practicing PCC in One’s Own Hospital.” Participants discuss current practice, challenges, and future initiatives (20 min each) to share practical perspectives.
60 min	Group Presentation and Discussion	Each group presents the outcomes of their discussion, followed by an open discussion to share learning.
60 min	Wrap-up	Summary and closing remarks on the training content.
Afternoon (30 min)	Post-Evaluation Survey	Post-intervention assessment using PCCI and TCCHI.

Note: ISO 9001 = International Organization for Standardization quality management systems standards; PCC = Person-centered care; PCCI = Person-Centered Care Instrument for Healthcare Providers; TCCN = Technological Competency as Caring in Nursing; TCCHI = Perceived Technological Competency as Caring in Healthcare Providers Instrument.

**Table 2:** Demographic profile of the nurse managers (*n* = 30).

Items	Category	*n* (%)
Age (in years)	20–29	1 (3.3)
	30–39	9 (30.0)
	40–49	11 (36.7)
	50–59	9 (30.0)
Length of Clinical Experience (in years)	5–9	2 (6.7)
	10–19	12 (40.0)
	20–29	8 (26.7)
	30–39	8 (26.7)
Highest Nursing Education	Nursing School/Diploma	7 (23.3)
	4-Year University (Bachelor)	7 (23.3)
	Master’s Degree	16 (53.3)
	Doctoral Degree	0 (0.0)
Current Institutional Position	Chief Nursing Officer/Director	5 (16.7)
	Deputy Director/Assistant CNO	4 (13.3)
	Head Nurse/Nurse Manager	9 (30.0)
	Charge Nurse/Supervisor	1 (3.3)
	In-Service Nurse Educator	3 (10.0)
	Nurse Specialist	6 (20.0)
	Senior Nurse	2 (6.7)
Geographical Region	Ulaanbaatar Metropolitan Area	17 (56.7)
	Non-Metropolitan/Rural Regions	13 (43.3)

Note: Percentages may not sum to 100.0% due to rounding. CNO = Chief Nursing Officer. Each participant is listed once, under their principal institutional designation. “In-Service Nurse Educator” and “Nurse Specialist” denote nurses who hold one of the four nursing management positions defined in Section 2.1 and who additionally carry designated in-service education or clinical-specialist responsibilities. “Senior Nurse” denotes the two participants who had been formally nominated for imminent appointment to a managerial position.

**Table 3:** Comparison of nurses’ self-reported PCCI and TCCHI scores before and after the one-day training program (*n* = 30).

Instruments/Concepts	Pre-Intervention Median (IQR)	Post-Intervention Median (IQR)	Score Changes (Inc/Dec/Unch), *n* (%)	*z*	*p*	*Adj. p*	*r*	Median Change (95% CI)
PCCI								
Total Score 37 items (37–222)	177.5 (24.50)	188 (32.25)	21 (70.0)/8 (26.7)/1 (3.3)	−2.98	0.003	0.023	0.54	+22.0 (7.0 to 35.5)
C1: Respect and Empathy 4 items (4–24)	20 (2.75)	21 (4.00)	17 (56.7)/7 (23.3)/6 (20.0)	−2.56	0.010	0.041	0.47	—
C2: Partnership and Trust 5 items (5–30)	24 (3.75)	25.5 (4.75)	20 (66.7)/6 (20.0)/4 (13.3)	−3.05	0.002	0.023	0.56	—
C3: Individualization/Diversity 4 items (4–24)	20 (3.75)	20 (3.00)	18 (60.0)/6 (20.0)/6 (20.0)	−3.32	< 0.001	0.014	0.61	—
C4: Shared Decision-Making 6 items (6–36)	28.5 (5.00)	30 (5.75)	19 (63.3)/9 (30.0)/2 (6.7)	−2.47	0.013	0.041	0.45	—
C5: Emotional and Psychological Support 4 items (4–24)	19 (3.00)	20.5 (3.00)	21 (70.0)/6 (20.0)/3 (10.0)	−3.03	0.002	0.023	0.55	—
C6: Comprehensive Care/Holistic Perspective 4 items (4–24)	19 (4.00)	20 (3.00)	18 (60.0)/5 (16.7)/7 (23.3)	−2.82	0.005	0.024	0.51	—
C7: Effective Information Sharing 6 items (6–36)	30 (5.75)	30 (4.00)	18 (60.0)/6 (20.0)/6 (20.0)	−2.45	0.014	0.041	0.45	—
C8: Flexible Care 4 items (4–24)	18.5 (3.00)	20 (3.00)	21 (70.0)/5 (16.7)/4 (13.3)	−3.17	0.002	0.019	0.58	—
TCCHI								
Total Score 38 items (38–228)	172.5 (28.50)	192 (33.00)	22 (73.3)/7 (23.3)/1 (3.3)	−3.17	0.002	0.019	0.58	+24.0 (10.5 to 38.0)
C1: Promoting self-growth 6 items (6–36)	28 (5.50)	30 (4.75)	23 (76.7)/6 (20.0)/1 (3.3)	−3.19	0.001	0.019	0.58	—
C2: Building trust relationship 7 items (7–42)	33.5 (4.75)	35.5 (4.50)	20 (66.7)/7 (23.3)/3 (10.0)	−2.96	0.003	0.023	0.54	—
C3: Providing PCC (Technology) 6 items (6–36)	27 (4.00)	30 (4.75)	23 (76.7)/6 (20.0)/1 (3.3)	−3.21	0.001	0.019	0.59	—
C4: Enhancing physical/emotional comfort 6 items (6–36)	26 (5.00)	31 (6.50)	19 (63.3)/7 (23.3)/4 (13.3)	−3.04	0.002	0.023	0.56	—
C5: Promoting patient learning/growth 6 items (6–36)	26 (5.75)	30 (5.00)	20 (66.7)/7 (23.3)/3 (10.0)	−3.28	0.001	0.015	0.60	—
C6: Engaging in ethico-moral practice 7 items (7–42)	32.5 (6.75)	35 (4.75)	17 (56.7)/9 (30.0)/4 (13.3)	−2.27	0.023	0.041	0.41	—

Note: Under each instrument and concept name, the number of items and the possible score range are given; each concept score is the simple sum of the scores of its items, each rated from 1 to 6. Median change (95% CI) = Hodges–Lehmann median of the within-person differences with a distribution-free 95% confidence interval, reported for the two primary outcomes only. C = concept; IQR = interquartile range; PCCI = Person-Centered Care Instrument for Healthcare Providers; TCCHI = Perceived Technological Competency as Caring in Healthcare Providers Instrument; Dec = decreased post-intervention score; Inc = increased post-intervention score; Unch = unchanged post-intervention score. *p* = unadjusted Wilcoxon signed-rank *p*-value; *Adj. p* = Holm-Bonferroni-adjusted *p*-value, controlling the family-wise error rate across all 16 comparisons; *r* = effect size for the Wilcoxon signed-rank test, Rosenthal’s r (*r* = z/√*N*), with *r* ≥ 0.30 conventionally interpreted as a moderate effect and *r* ≥ 0.50 as a large effect [36,37]. For concept 3 (PCCI), the pre- and post-intervention medians are identical despite the statistically significant result; see main text (Section 3.2.1) for interpretation. In the present sample (*n* = 30), the internal consistency of the total scales was acceptable for both the Mongolian PCCI (*ω* = 0.979, *α* = 0.969) and TCCHI (*ω* = 0.968, *α* = 0.790). These coefficients describe internal consistency within the present sample only and do not constitute evidence that the Mongolian versions have been validated.

## Data Availability

The de-identified, individual-level dataset supporting the findings of this study (anonymized pre- and post-intervention PCCI and TCCHI item and total scores for the *n* = 30 analytic sample, linked only by non-identifying code number) is available from the corresponding author upon reasonable request. These data are not deposited in a public repository because the study sample is small and drawn from a single, identifiable symposium cohort in Mongolia such that even de-identified individual-level data could carry a risk of re-identification; the Tokushima University Hospital Ethics Committee approval (no. 4708) did not include provisions for public data deposition. Requests will be reviewed by the corresponding author and, where necessary, in consultation with the Mongolian collaborating institutions. The ethics-approved study protocol (Tokushima University Hospital, approval no. 4708; Mongolia-Japan Hospital, MNUMS, approval no. A/08-2) is also available from the corresponding author on reasonable request. The educational materials used in the program (lecture materials, slides, and the workshop facilitation guide) are not included in this publication and remain the copyright of the authors; they may be made available by the corresponding author on reasonable request for non-commercial academic or educational use, subject to a written agreement covering attribution and restrictions on redistribution, adaptation, and translation. Requests may be declined or deferred where the materials are being prepared for separate publication or development as a teaching resource. Two departures from the approved protocol are noted. First, the sensitivity analysis excluding the two participants who had been nominated for, rather than currently holding, a managerial position was not specified in the protocol and was added in response to peer review. Second, the Hodges–Lehmann change estimates with 95% confidence intervals reported for the two primary outcomes were added during peer review at the editor’s request and were not specified in the protocol. No changes were made to the eligibility criteria, the intervention, or the designation of the primary outcomes after the protocol was approved.

## Supplementary Materials

The following supporting information can be downloaded at: https://www.mdpi.com/article/10.3390/nursrep16090320/s1, Table S1. Sensitivity analysis: comparison of self-reported PCCI and TCCHI scores before and after the one-day training program, excluding the two participants who had been formally nominated for, rather than currently holding, a managerial position (*n* = 28).

## Public Involvement Statement

No members of the public were involved in the design, conduct, or reporting of this research.

## Guidelines and Standards Statement

This study was reported in accordance with the Transparent Reporting of Evaluations with Nonrandomized Designs (TREND) statement for non-randomized evaluations of behavioral and public health interventions.

## Use of Artificial Intelligence

AI-assisted tools (Claude, Anthropic PBC, San Francisco, CA, USA) were used in the preparation of this manuscript for English language editing, for verifying and recomputing the statistical analyses from the authors’ own dataset, for drafting and revising sections of the text. Figure 1 was created using matplotlib (version 3.10.8) for Python. All AI-assisted output was checked and verified by the authors, who take full responsibility for the content of the manuscript, the accuracy of the data, and the interpretation of the findings.

## Abbreviations

The following abbreviations are used in this manuscript:

CNO	Chief nursing officer
CI	Confidence interval
IQR	Interquartile range
ISO 9001	International Organization for Standardization 9001 quality management systems standard
JICA	Japan International Cooperation Agency
LMICs	Low- and middle-income countries
MNUMS	Mongolian National University of Medical Sciences
PCC	Person-centered care
PCCI	Person-Centered Care Instrument for Healthcare Providers
TCCHI	Perceived Technological Competency as Caring in Healthcare Providers Instrument
TCCN	Technological Competency as Caring in Nursing
TCTR	Thai Clinical Trials Registry
TREND	Transparent Reporting of Evaluations with Nonrandomized Designs
