# Supplementary material for: Immediate Changes in Perceived Person-Centered Care and Technological Competency Following a One-Day Collective Training Program Among Mongolian Nurse Managers and Nurses Nominated for Managerial Roles: A Pilot Pre–Post Study
Source: Nurs Rep. 2026 Sep 4;16(9):320. doi: 10.3390/nursrep16090320 (PMC13609953; doi:10.3390/nursrep16090320)
Supplement: Supplementary file 1 [file nursrep-16-00320-s001.zip › nursrep-4435626-supplementary.pdf]

Supplementary Table S1. Sensitivity analysis: comparison of self-reported PCCI and TCCHI scores before and after the one-day training program, excluding the two participants who had been formally nominated for, rather than currently holding, a managerial position (*n* = 28)

**Instruments / Concepts**	**Pre-intervention Median (IQR)**	**Post-intervention Median (IQR)**	**Score Changes (Inc / Dec / Unch), *n* (%)**	***z***	***p***	***Adj. p***	***r***
**PCCI**							
Total Score	175.5 (23.75)	190.5 (33.50)	20 (71.4) / 7 (25.0) / 1 (3.6)	-3.14	0.002	0.013	0.59
C1: Respect and Empathy	20.0 (3.25)	21.0 (4.00)	16 (57.1) / 6 (21.4) / 6 (21.4)	-2.59	0.010	0.019	0.49
C2: Partnership and Trust	24.0 (4.00)	25.5 (5.00)	19 (67.9) / 5 (17.9) / 4 (14.3)	-3.21	0.001	0.013	0.61
C3: Individualization/Diversity	19.5 (4.00)	20.5 (3.25)	18 (64.3) / 4 (14.3) / 6 (21.4)	-3.45	< 0.001	0.008	0.65
C4: Shared Decision-making	28.0 (5.25)	30.0 (6.00)	19 (67.9) / 7 (25.0) / 2 (7.1)	-2.96	0.003	0.015	0.56
C5: Emotional and Psychological Support	19.0 (3.25)	20.5 (3.25)	20 (71.4) / 5 (17.9) / 3 (10.7)	-3.15	0.002	0.013	0.59
C6: Comprehensive Care/Holistic Perspective	18.5 (4.25)	20.0 (3.25)	18 (64.3) / 4 (14.3) / 6 (21.4)	-3.04	0.002	0.014	0.57
C7: Effective Information Sharing	30.0 (5.50)	30.0 (4.25)	17 (60.7) / 5 (17.9) / 6 (21.4)	-2.55	0.011	0.019	0.48
C8: Flexible Care	18.0 (3.00)	20.0 (3.25)	20 (71.4) / 4 (14.3) / 4 (14.3)	-3.21	0.001	0.013	0.61
**TCCHI**							
Total Score	171.5 (30.00)	194.0 (35.25)	21 (75.0) / 6 (21.4) / 1 (3.6)	-3.39	< 0.001	0.010	0.64
C1: Promoting self-growth	27.5 (4.50)	30.5 (5.50)	22 (78.6) / 5 (17.9) / 1 (3.6)	-3.25	0.001	0.013	0.61
C2: Building trust relationship	33.5 (4.25)	35.5 (4.50)	19 (67.9) / 6 (21.4) / 3 (10.7)	-2.90	0.004	0.015	0.55
C3: Providing PCC (Technology)	27.0 (4.25)	30.5 (5.00)	22 (78.6) / 5 (17.9) / 1 (3.6)	-3.50	< 0.001	0.007	0.66
C4: Enhancing physical/emotional comfort	26.0 (5.00)	31.0 (6.50)	19 (67.9) / 5 (17.9) / 4 (14.3)	-3.27	0.001	0.013	0.62
C5: Promoting patient learning/growth	26.0 (5.25)	30.0 (5.00)	19 (67.9) / 6 (21.4) / 3 (10.7)	-3.33	< 0.001	0.011	0.63
C6: Engaging in ethico-moral practice	32.0 (7.25)	35.0 (4.25)	17 (60.7) / 7 (25.0) / 4 (14.3)	-2.86	0.004	0.015	0.54

Note. C = concept; IQR = interquartile range; PCCI = Person-Centered Care Instrument for Healthcare Providers; TCCHI = Perceived Technological Competency as Caring in Healthcare Providers Instrument; Inc = increased post-intervention score; Dec = decreased post-intervention score; Unch = unchanged post-intervention score. p = unadjusted Wilcoxon signed-rank p-value; Adj. p = Holm–Bonferroni-adjusted p-value, controlling the family-wise error rate across all 16 comparisons; r = effect size for the Wilcoxon signed-rank test ($r = z/\sqrt{N}$; Rosenthal's r), with N = 28. Zero differences were excluded and average ranks were used for ties; z was computed with tie correction and the two-tailed p-value from the normal approximation. This sensitivity analysis was not pre-specified and was conducted in response to peer review. All 16 comparisons remained statistically significant after correction (all adjusted $p \leq 0.019$), and the effect sizes (0.48–0.66) were comparable to those obtained in the full sample of 30 participants (0.41–0.61).
